# Correlations of Theory of Mind Deficits with Clinical Patterns and Quality of Life in Schizophrenia

**DOI:** 10.3389/fpsyt.2013.00030

**Published:** 2013-05-06

**Authors:** Mathieu Urbach, Eric Brunet-Gouet, Nadine Bazin, Marie-Christine Hardy-Baylé, Christine Passerieux

**Affiliations:** ^1^ECIPSY EA4047, Faculté de Médecine, Université de Versailles-Saint QuentinVersailles, France; ^2^Pôle de Psychiatrie, Centre Hospitalier de VersaillesLe Chesnay, France

**Keywords:** schizophrenia, theory of mind, symptoms, quality of life

## Abstract

**Background:** Numerous studies have demonstrated the existence of theory of mind (ToM) impairments in patients with schizophrenia. The clinical consequences of these impairments are currently under debate. Accumulated evidence suggests that ToM deficits are linked to negative and disorganization symptoms, but direct correlations are lacking. Moreover, it is unclear whether ToM deficits are related to reduced quality of life (QoL).

**Methods:** To extend the understanding of objective (i.e., clinical symptoms) and subjective (QoL) correlates of impaired ToM, we assessed 206 patients with schizophrenia based on performance of an ecological task (Versailles-Situational Intention Reading, V-SIR), a Communication Disorders Scale (SCD), the Positive and Negative Syndrome Scale (PANSS), the Clinical Global Impression rating, and a QoL questionnaire (S-QoL). Statistical inferences were drawn from correlations analyses considering both factors/subscales aggregates and single items.

**Results:** ToM performance was negatively correlated to disorganization and negative PANSS factors. Poor V-SIR performance was correlated with “conceptual disorganization,” “difficulties in abstract thinking,” and “apathy/social withdrawal.” The SCD was correlated with “negative,” “disorganization,” and “anxiety/depression” PANSS factors. The S-QoL total score was not significantly correlated with ToM performance. Only the item “difficulties in expressing feelings” was significantly correlated with poorer V-SIR performance.

**Conclusion:** We discuss the intriguing paucity of the results and what they reveal about the difficulties faced by psychiatrists with patients not expressing complaints about lack of social skills.

## Introduction

Within the domain of social cognition, “theory of mind” (ToM), or “mentalizing,” refers to the ability to infer and to imagine one’s own and another person’s mental states; i.e., thoughts, beliefs, intentions, and knowledge. In the affective domain, these skills participate in understanding another person’s feelings and emotions, and more generally to empathize with others. According to Frith (1992), schizophrenia is characterized by profound impairments in the cognitive mechanisms that underpin ToM. In agreement with this view, many results, previously summarized in two meta-analyses, demonstrated that individuals with schizophrenia have significantly reduced performance in several ToM subdomains compared to healthy controls (Sprong et al., 2007; Bora et al., 2009). In comparison with healthy subjects, the patients have reduced performances with large effect sizes for the first order false belief (*d* = 1.19), second order false belief (*d* = 1.44), indirect speech understanding (*d* = 1.04), and intention inference (*d* = 0.96) (Sprong et al., 2007). It is worth noting that these impairments were found to be equally or more severe than deficits in neuropsychological skills, including memory, attention, and executive functions (see meta-analysis, by Heinrichs and Zakzanis, 1998).

In considering the functional significance of cognitive impairments, deficits in ToM may hypothetically play a role in the psychopathology of schizophrenia and be apparent within the large set of schizophrenic symptoms. Disorganization and negative symptoms seem the more probable expression of ToM impairments. According to the neo-Bleulerian approach, it was previously hypothesized that disorganization syndrome (i.e., disorganized thinking, communication, and behavior) could be caused by impaired ToM skills and impaired processing of contextual information (Hardy-Baylé et al., 2003; Green et al., 2005). That view received some confirmation from studies that used a non-verbal paradigm, based on comic-strips, to show that the attribution of intentions to others is particularly impaired in patients with schizophrenia that exhibited formal thought disorders (Sarfati et al., 1997; Brüne, 2005). Abdel-Hamid et al. (2009) provided direct evidence of the relationship between the Positive and Negative Syndrome Scale (PANSS, Kay et al., 1987) “disorganization” factor and ToM deficits in a correlation analysis performed on 50 patients with schizophrenia. Finally, with the power conferred from a meta-analytic procedure, Sprong et al. (2007) demonstrated that patients with disorganization symptoms had higher ToM deficits than patients that lacked disorganization symptoms, were paranoid, or had remitted. The link between negative symptoms and impaired ToM has been hypothesized because of the consequences of both in social life. It has been found that schizophrenic patients with “negative behavioral” clinical signs had the poorest performances in different ToM tasks (Corcoran et al., 1995, 1997; Pickup and Frith, 2001). Association between ToM impairment and negative symptoms was also found when using PANSS subscale (Lincoln et al., 2011). Yet the former review of Sprong et al. had not found an association with negative symptoms as with disorganization. The reason may be the lack of specific assessment of this dimension, as provided by Lincoln et al. in their study. To date, there is still a quite imprecise knowledge on the subtle clinical expression of ToM impairments in schizophrenia. The present study will add to this knowledge by considering the patient’s clinical status at both the syndrome (by following a factor analysis procedure) and the item levels.

Obviously, another consequence of social cognitive disorders should be profound impairment of social life and functioning. Firstly, a meta-analysis based on over 50 studies, considering “objective” measures of functioning, demonstrated the weight of social cognition as a predictor of functional outcome (Fett et al., 2011). It is now acknowledged that both social cognition and neurocognition influence the patient’s ability to achieve social roles within the contexts of family, friendship, work, or occupation. Secondly, quality of Life (QoL) tools are another way to measure a patient’s functioning requiring the subjective assessment of his/her situation considering the main domains of everyday life. QoL corresponds to the patient’s satisfaction in personal, professional, or occupational domains and allow a specific view on her or his skills, distinct from assessments of real-life functioning.

Hypothetically, ToM deficits should be involved when patients describe a difficult and/or an impoverished social life and reduced satisfaction in human relationships. To our knowledge, only one study has documented a relationship between ToM performance and the satisfaction expressed by patients with their life (Maat et al., 2012). In that study, a sample of 1032 patients was compared with 1011 siblings and 552 healthy controls. Those authors demonstrated that only patients with schizophrenia exhibited a significant relationship between QoL and performance on the hinting task (a verbal ToM task assessing the attribution of intentions behind indirect utterances). Moreover, that relationship was influenced by symptoms; a significant negative correlation was found between QoL and high ToM performance in patients with relatively high symptom levels. The results suggested that a poor QoL could reveal overall symptom consequences on the patient’s life, and these consequences might be influenced by ToM skills, because this is related to insight (Bora et al., 2007). While these findings are extremely relevant, replication and refinement of the data description are needed.

The goal of the present study was to investigate clinical symptoms and QoL items to determine which would reveal an underlying ToM impairment in patients with schizophrenia. We assessed ToM with two tasks: an ecological task based on videos assessing the attribution of intentions to others (Versailles-Situational Intention Reading, V-SIR; Bazin et al., 2009) and the Communication Disorders Scale (SCD), a semi-structured interview, which assessed both communication skills and the ability to attribute mental states to oneself or to others under conversational constraints (Bazin et al., 2005). A strength of this study is the use of these two tools based on very different experimental methods to assess overlapping social cognitive constructs. Our first hypothesis was that clinical symptoms pertaining to negative and disorganization syndromes would exhibit the strongest correlations with ToM deficits. The second hypothesis was that we would find significant correlations between ToM impairments and QoL items that concerned social life and interpersonal relationships. Based on the findings of Maat et al. (2012), our third hypothesis was that QoL would be predicted by overall symptoms (PANSS total), influenced by an interaction effect with ToM skills.

## Materials and Methods

### Study population

The main study population consisted of 281 patients with schizophrenia (85 females, 196 males) that had been recruited during the baseline of a pharmaceutical protocol that included an antipsychotic medication switch. Inclusion criteria were: (1) aged 18–65 years, (2) exhibited criteria for schizophrenia based on the Diagnostic and Statistical Manual of Mental Disorders, Fourth Edition (DSM-IV), (3) taking a stable dose of a single antipsychotic therapy for at least 8 weeks, and (4) PANSS score above 45. A seen in the result section, a population of 206 patients was kept in the final analysis.

This study was part of a multicenter, pharmacological, longitudinal trial conducted and supported by Lilly France (“Outcome” study, F1D-FP-S029). The authors had full access to all data required for the investigative use of the V-SIR and SCD tools, but remained blind regarding the study treatments (i.e., no information regarding study treatments was provided to the authors) and to some sociodemographic data, including occupation. All participants gave informed consent to participate in this study. The local ethics committee (Comité de Protection des Personnes participant à une Recherche Biomédicale) approved this research (June 2003) in agreement with the international standard for the conduct of human research.

### Measures

Senior psychiatrists and psychologists having followed videotaped training for rating all the instruments used in this protocol evaluated the patients.

#### Clinical assessment

##### Positive and negative syndrome scale

The PANSS allowed a complete, standardized evaluation of psychotic symptoms (Kay et al., 1987). The scale has 7 positive-symptom items, 7 negative-symptom items, and 16 general psychopathology symptom items. Each item is scored on the same seven-point severity scale, from 1 (normal) to 7.

##### Clinical global impression

The Clinical Global Impression Severity (CGI-S) rating was employed in the clinician’s global evaluation of illness severity (Guy, 1976). The CGI-S provided an evaluation of severity on a seven-point scale, with a range from 1 (normal) to 7 (severely ill).

#### Theory of mind evaluation

##### Scale for the evaluation of communication disorders

The Scale for the evaluation of Communication Disorders (SCD) was used to provide an assessment of communication disorganization and the ability to understand mental states, i.e., the ToM (Olivier et al., 1997). A validation study (Bazin et al., 2005) had demonstrated that this evaluation was highly specific for schizophrenia, with respect to mania and depression, showed good sensitivity to change, and was highly correlated (*r* = 0.66) with the Scale for the Assessment of Thought, Language, and Communication (Andreasen, 1986).

It consisted of a semi-structured interview performed by a trained clinician. The SCD provided conversational and cognitive constraints to elicit erroneous or disorganized speech. Six tasks were evaluated, and each corresponded to a category of pragmatic constraints. The six tasks were: (1) to summarize a speech corpus; (2) to attribute intentions to others; (3) to describe the clinician’s intention during the interview; (4) to attribute an intention to one’s own speech; (5) to process a semantic ambiguity; and (6) to attribute an erroneous belief to a character in a short story. Evaluations were based on a four-level scale (0 – absent to 3 – severe), thus ranging from 0 (no disorganization) to 21 (extreme disorganization).

##### Versailles-situational intention reading

Theory of mind was also assessed with the V-SIR, which offered a good compromise between ecological validity (real life, interpersonal situations are depicted) and ease of deployment in a multicenter study (Bazin et al., 2009). The subjects were asked to evaluate the likelihood of propositions concerning the intentions of characters involved in short video excerpts that showed complex social interactions. V-SIR video material consisted of six scenes, each 10–60 s long. The same videos in the same order were presented to every subject. The scenes showed complex, realistic interactions between two characters or more and involved hints, lies, indirect speech etc. The patient had to perceive social cues (prosody, gestures, speech, facial emotional display…) and interpret them correctly to be able to attribute the appropriate mental states to the characters. After each video, five interpretations were proposed to the subject. Each interpretation suggested a hypothetical explanation of one character’s behavior. The proposals were designed to elicit common errors of interpretation and reasoning biases; for instance, each video was associated with one absurd interpretation, and with another interpretation commonly widespread, but faulty in the presented context. An explicit formulation (“because,” “in order to” etc…) was used to signify that the question clearly addressed the character’s intention. The subject rated independently each interpretation as “very unlikely,” “unlikely,” “likely,” or “very likely.” No choice among these interpretations was required. After a 10-min training session, the task was conducted over approximately 20–30 min.

A score was calculated by attributing a value to each response (“very unlikely” = 1, “unlikely” = 2, “likely” = 3, “very unlikely” = 4) and by comparing it to the average of the responses of a control group. A single subject’s total score was computed as the sum of the absolute values of the differences between the subject’s ratings of each item and the mean ratings of the control group. The higher a score, the more the subject is impaired in ToM.

#### Quality of life (S-QoL 41)

The S-QoL 41 is a self-administered scale designed to assess health-related QoL in people with schizophrenia. It is worth noting that the S-QoL was designed to evaluate only the satisfaction of the subject, and not the performance level. The validation study, involving 207 patients, showed high internal consistency, reliability, reproducibility, and responsiveness, and it confirmed the validity of the S-QoL construct (Auquier et al., 2003). Satisfaction was assessed through 41 items, clustered in eight subscales (psychological well-being, self-esteem, family, relationships, relationships with friends, resilience, physical well-being, autonomy, and sentimental life) and a total score. Each items was accompanied by a five-point scale, from 1 = “less than expected” to 5 = “more than expected.” For each subject, the score of each of the eight dimensions was obtained by computing the mean of the item scores of the dimension. All scales were linearly transformed to a 0–100 scale, with 100 indicating the most favorable QoL, and 0 the least favorable.

### Statistical analyses

The statistical analyses were designed straightforwardly to explore whether patient characteristics were related to ToM performance (evaluated through V-SIR and SCD). For hypotheses I and II, we explored the data with Pearson’s correlation coefficients. Relationships were tested between ToM and subjects’ age, the CGI-S, the PANSS, or the S-QoL. Syndrome analyses were performed with a factor analysis on PANSS items. A varimax rotation was used to introduce the five factors into the model (this number was set empirically, based on the literature; Van den Oord et al., 2006). Correlations with these factors were tested. To prevent type I errors, we adopted a stringent 0.001 threshold for all correlation coefficients examined. To assess the third hypothesis, we used a stepwise linear regression procedure that introduced the total S-QoL as the response variable and age, gender, V-SIR, SCD, and PANSS as the predictors following Maat et al.’s (2012) design. We also introduced the interaction terms, PANSS*V-SIR and PANSS*SCD, into the model. Throughout the article, results are expressed as the mean ± standard deviation, unless otherwise noted.

## Results

### Sample characteristics

A total of 149 men and 57 women were included, with mean ages of 40.7 ± 10.3 and 44.7 ± 10.0 years, respectively. Seventy-five patients were excluded from the analysis due to missing data (among whom 30 patients were not administered the V-SIR and 33 had more than one missing item). Unpaired *t*-statistics were used to compare age, sex, CGI-S, and each PANSS item between the excluded and included patients. No significant difference was found with a 0.05 threshold, except in the “active social withdrawal” PANSS item. It was difficult to take this difference into account in subsequent analyses, because the included patients exhibited higher scores for this item than the excluded patients (mean ± standard deviations: 3.1 ± 1.3 and 2.7 ± 1.4, respectively; *P* = 0.035).

### Clinical characteristics (PANSS and CGI)

Clinical (CGI-S, PANSS) evaluations are presented in Table 1. A five-dimensional factor analysis of PANSS items with varimax rotation exhibited a structure similar to that expected from the literature (Van den Oord et al., 2006). When load scores were above 0.4, correlations between scores for PANSS items and factors were computed and the following names were assigned to factors: positive, negative, disorganization, anxiety/depression, and excitation/persecution (see items repartition into factors in Table 2).

**Table 1:** **Clinical and social cognitive data of the patients**.

	Mean (standard deviation)	Min value	Max value
CGI-S	4.16 (0.92)	2	6
PANSS total score	79.4 (16.34)	45	140
PANSS positive score	16.46 (5.15)	7	33
PANSS negative score	23.19 (5.68)	10	43
PANSS general score	39.75 (9.77)	18	76
V-SIR	24.32 (7.82)	9.4	49.19
SCD total score	7.6 (4.1)	0	18

**Table 2:** **Correlation analysis between V-SIR and SCD scores and PANSS items, classified with respect to a five-dimensional factor analysis with the exception of the two last items**.

PANSS item	*r* V-SIR	*r* SCD	Loading score^a^
**FACTOR 1: “NEGATIVE”**			
Motor retardation	0.1	0.04	0.56
Lack of spontaneity	0.12	0.19	0.55
Active social avoidance	0.14	0.21	0.51
Blunted affect	0.15	0.24*	0.7
Poor rapport	0.19	0.34*	0.66
Emotional withdrawal	0.21	0.31*	0.83
Apathy, social withdrawal	0.23*	0.23*	0.75
Difficulties of abstract thinking	0.35*	0.33*	0.44
**FACTOR 2: “DISORGANIZATION”**			
Stereotyped thinking	0	0.21	0.46
Disturbance of volition	0.07	0.24*	0.49
Disorientation	0.08	0.09	0.44
Lack of judgment	0.12	0.26*	0.42
Poor attention	0.17	0.19	0.45
Conceptual disorganization	0.25*	0.42*	0.5
**FACTOR 3: “ANXIETY/DEPRESSION”**			
Anxiety	−0.08	−0.03	0.66
Guilt feelings	−0.04	−0.13	0.55
Tension	−0.03	−0.03	0.77
Depression	0.01	−0.06	0.47
Somatic concern	0.03	0.1	0.41
**FACTOR 4: “POSITIVE”**			
Grandiosity	0.01	0.09	0.45
Hallucinatory behavior	0.03	0.01	0.66
Unusual thought	0.04	0.16	0.63
Delusions	0.09	0.15	0.88
**FACTOR 5: “EXCITATION/PERSECUTION”**			
Hostility	0	0	0.81
Excitement	0.01	0.01	0.47
Suspiciousness	0.01	−0.01	0.47
Poor impulsive control	0.02	0.06	0.5
Un-cooperativeness	0.07	0.15	0.42
**NOT AFFECTED TO A FACTOR**			
Preoccupation	0.01	0.08	–
Mannerisms	0.02	0.15	–

*r* V-SIR, Pearson’s correlation coefficient for Versailles-situational intention reading test score; *r* SCD, Pearson’s correlation coefficient for scale for the evaluation of communication disorders score. ^a^Loading scores for each factor are given when above 0.4. *P ≤ 0.001.

### Theory of mind assessment (V-SIR, SCD)

Table 1 indicates the main results concerning V-SIR and SCD. Let’s note that the V-SIR scores followed a normal distribution (Kolmogorov–Smirnov test *d* = 0.06; *P* > 0.20). The distribution of each item ranged from the minimum possible score (1) to the maximum score (4).

We found no statistically significant influence of gender on the V-SIR score (*t*-test, *t* = −0.69, *P* = 0.49). The V-SIR score correlated positively with age (*r* = 0.21; *P* < 0.05). A significant correlation between V-SIR and summed SCD scores was found (*r* = 0.41; *P* < 0.0001). Taken separately, each SCD item correlated significantly with V-SIR (*r* > 0.24; *P* < 0.001). The inspection of the correlations between V-SIR and SCD items showed that the most significant correlations are found with “Difficulties in attributing intentions to others” and “Inability to process a semantic ambiguity” (Table 3).

**Table 3:** **Correlations between V-SIR and SCD items**.

SCD items	*r* V-SIR
Inability to summarize a speech corpus	0.34*
Difficulties in attributing intentions to others	0.42*
Difficulties in describing the clinician’s intention during the interview	0.32*
Difficulties in attributing an intention to one’s own speech	0.23*
Inability to process a semantic ambiguity	0.41*
Inability to attribute an erroneous belief to a character in a short story	0.30*

Pearson correlation coefficients are shown. *P < 0.001.

### Relationship of V-SIR and SCD with CGI and PANSS

The CGI-S scores correlated significantly with V-SIR (*r* = 0.26; *P* < 0.001) and SCD total scores (*r* = 0.32; *P* < 0.001).

Correlations were tested between V-SIR, SCD, and the five factors obtained from the varimax rotation factor analysis of PANSS items (Table 4). V-SIR correlated significantly with the “negative” factor (*r* = 0.24, *P* < 0.001), and a trend was found with the “disorganization” factor (*r* = 0.013, *P* = 0.057). No significant correlations were found with the “anxiety/depression,” “excitation/persecution,” or “positive” factors (*P* > 0.05). SCD total scores exhibited significant correlations with the “negative” factor (*r* = 0.33; *P* < 0.0001) and the “disorganization” factor (*r* = 0.28; *P* < 0.0001). A significant negative correlation was found with the “anxiety/depression” factor (*r* = −0.17; *P* < 0.05). No significant correlation was found with the “positive” or “excitation/persecution” factor (*P* > 0.05).

**Table 4:** **Correlation analysis between V-SIR and SCD scores and PANSS factors**.

PANSS factor	*r* V-SIR	*r* SCD
Factor 1: “negative”	0.24 (0.00)	0.32 (0.00)
Factor 2: “disorganization”	0.13 (0.06)	0.27 (0.00)
Factor 3: “anxiety/depression”	−0.11 (0.10)	−0.17 (0.01)
Factor 4: “positive”	0.06 (0.36)	0.10 (0.13)
Factor 5: “excitation/persecution”	−0.00 (0.96)	−0.00 (0.98)

*Pearson correlation coefficients and corresponding *P* values*.

Table 2 shows the correlations between the PANSS items and the V-SIR and SCD scores. Although the coefficients for the V-SIR corresponded to small or medium effect sizes (Cohen, 1988), some reached the 0.001 significance level, including “conceptual disorganization,” “difficulties of abstract thinking,” and “apathy/social withdrawal.” The SCD was significantly correlated with “Blunted affect,” “Poor rapport,” “Emotional withdrawal,” “Disturbance of volition,” and “Lack of judgment.”

### Relationship of V-SIR and SCD items with quality of life scores

When correlation analyses with a 0.001 threshold were applied to S-QoL total scores and subscales, no significant correlations were revealed with either V-SIR or SCD scores. To avoid running the risk of a type I error due to multiple correlations, we report only a trend toward a negative correlation between V-SIR and “psychological well-being” (*r* = −0.14; *P* < 0.05).

An item-by-item analysis did not reveal significant correlations, except with the S-QoL item, “I have difficulties expressing my feelings,” which was correlated with V-SIR (*r* = −0.24; *P* < 0.001). This negative correlation, due to the scoring procedure, indicated that greater ToM deficits may lead to the report of greater difficulties in expressing one’s own emotions.

### Regression analysis of quality of life scores

A stepwise regression analysis was performed to determine whether S-QoL total score was associated with age, gender, PANSS total score, V-SIR, and SCD (degrees of freedom for error: 200; degrees of freedom for the regression: 1; total sum of squares of the response: 6.36*e* + 04; sum of squares of the residuals: 6.0*e* + 04; *F*-statistic for testing the final model vs. no model: 9.74; *P* value of the *F*-statistic: 0.0021; root mean square error: 17.41). This procedure showed that only the PANSS total score could significantly predict S-QoL (beta coefficient = −0.233 SD = 0.07, *P* = 0.002). No interaction terms (PANSS total*V-SIR or PANSS total*SCD) reached the 0.05 threshold for inclusion into the model.

## Discussion

The goal of the present study was to describe the panorama of relationships among the ToM (assessed with V-SIR and SCD), clinical symptoms (assessed with the PANSS and CGI-S), and QoL (assessed with the S-QoL 41) in a population of non-hospitalized, medicated patients with schizophrenia. In agreement with our first hypothesis, we found significant correlations between ToM deficits and both negative and disorganization syndromes. In contradiction to the second hypothesis, we found no correlation between ToM deficits and the S-QoL total score or subscores. Moreover, we found no significant interaction between ToM performance and clinical status that might have been used to predict S-QoL scores.

To begin with the relationships of ToM performances with demographic variables, the present study gives evidence that V-SIR score is correlated positively with age. The previous study of Bazin et al. (2009) found no significant correlation between this score and age in the schizophrenic group or in the depressed group, but a positive correlation in the manic patients group (*r* = 0.60, *P* = 0.02). However, this negative finding may be explained by the little number of schizophrenic patients recruited in this first work (*n* = 15). One possible interpretation of the present finding could be that the duration of the illness, which increases with age, has a negative influence on ToM performances as suggested by the meta-analysis from Bora et al. (2009). Further research addressing specifically this question would be necessary.

The data presented here allowed an exploratory analysis of the influence of gender on ToM performances. The interest of exploring such an effect in mental illnesses is grounded on the literature on child development and autism. In healthy preadolescents, it has been shown that girls have higher ToM and self-understanding performances than boys, independent of language skills (Bosacki, 2000). Baron-Cohen (2002) theorized the influence of gender on cognitive styles through the extreme-male theory of autism, a pathology where, putatively, patients exhibit a male’s tendency to systemize and analyze things within a system of lawful regularities rather than to empathize or identify others’ mental states. Concerning gender, our results suggest an absence of a significant gender effect on V-SIR performances in schizophrenic patients. As we do not have reference data using V-SIR with comparable healthy populations, it is not possible to establish whether this absence of gender effect is a specificity of schizophrenia or not.

### Relationship between ToM and symptoms

Our results brought to light positive correlations between ToM impairments, assessed with two ToM instruments (SCD and V-SIR), and PANSS factors that belonged to disorganization and negative syndromes. These results replicated the findings of Abdel-Hamid et al. (2009), who also showed a negative correlation between ToM performance and “Conceptual disorganization” or “Difficulty in abstraction.” However, those authors found only a weak association between ToM and negative symptoms. One interesting finding from the present study was the agreement between results obtained with two distinct ToM paradigms; the first was based on semi-structured interviews and enforcement of specific conversational constraints, and the second consisted of evaluations of potential assertions about video excerpts.

Interestingly, the SCD used in the present protocol lead to larger coefficients of correlation with V-SIR performances when compared with the PANSS. More precisely, the item “inability to process semantic ambiguity,” which does not require explicitly ToM inferences, exhibited the positive correlation with the largest magnitude (i.e., *r* = 0.41). It is useful to remind that the SCD was designed to overcome the difficulty of assessing specific clinical expression of schizophrenia such as thought and communication disorganization. Unlike the PANSS, this instrument consists of several methods which stress patients’ cognitive systems by imposing conversational constraints, such as summarizing one’s own speech, or explicitly questioning ones’ or others’ mental state (Brunet, 2007). Supposedly, the patients being placed under the appropriate cognitive load, focused at their cognitive deficits (i.e., impaired ToM), will exhibit abnormal and/or disordered speech and thought. It is striking that such a way of performing a clinical evaluation, avoiding direct symptom assessment by the means of a structured interview focused on living habits and conditions, has exhibited the highest correlation with V-SIR ToM.

No significant relation was found between ToM impairments and the positive syndrome, which encompasses hallucinations, delusions, and passivity symptoms. This syndrome has been conceptualized as a consequence of a “metarepresentation disorder,” which was thought to be a key mechanism underlying ToM skills (Frith, 1992). However, studies gave contradictory results. In a meta-analysis, Sprong et al. (2007) reported the existence of ToM impairments in patients with paranoia, but a stronger association was found in other subgroups; i.e., between ToM and the subgroup of patients with disorganization syndrome. The contrasting results on this point may be due to differences in the methodology. In a video-based task study, which included 58 patients with schizophrenia-spectrum disorders that exhibited acute persecutory delusions, these symptoms were associated with a reduced ability to infer intentions compared with patients in remission (Mehl et al., 2010). With a larger sample, Abdel-Hamid et al. (2009) reported the absence of a significant correlation between the PANSS positive items and ToM measures. It is worth noting that those differences cannot be attributed to the intensity of the positive symptoms: the PANSS positive subscale score was similar in Mehl et al. and in our study (respectively, 14.76 ± 4.7 and 16.46 ± 5.15). The present study added negative findings to these contrasting results on the positive symptoms; however, our results emphasized the relevance of evaluating ToM in patients with negative or disorganization syndromes.

### Relationship between theory of mind and quality of life

This study investigated the correlations between ToM measures and a QoL measure. To the best of our knowledge, few studies have investigated these links: only a recent study (Maat et al., 2012) showed a significant association between ToM and QoL. That study showed that the more skill the subjects showed in ToM, the less they reported satisfaction in their situation. Moreover, those authors demonstrated a significant interaction between ToM performance and symptoms; thus, in patients with preserved ToM, illness severity was correlated with the reported QoL. The present study did not replicate that interaction; we found that the S-QoL global score was predicted by the PANSS total score, but not by ToM performance.

This apparent contradiction in results may be due to population and/or methodological differences. The two populations may not be similar: when using the same way to calculate the PANSS score, we found a higher mean “total score” than Maat et al. (2012) (8.14, SD 1.62 vs. 5.54, SD 1.7). It is also possible that the large sample size in the Maat et al. study (1032 vs. 206 in the present study) facilitated the emergence of significant statistical effects that the current study could not discern, due to the lower statistical power. However, this is unlikely, because the magnitude of the interaction effect was considered “large” by those authors (*f*^2^ = −0.256); thus, it should have been readily replicated with a sample smaller than that of the present study. The tools used to assess QoL and ToM are different in the two studies. Maat et al. used the WHOQOL-BREF scale, which was not specifically designed for patients with schizophrenia, and it did not assess exactly the same domains as assessed by the S-QoL. The WHOQOL-BREF assessed the level of satisfaction, but the S-QoL, inspired by interviews with schizophrenic patients rated the discrepancy between the patient’s actual state and his/her expectation (Auquier et al., 2003). Thus, the point of view of the WHOQOL-BREF may be the one of the psychiatrist more than the one of the patient. Yet this last approach has proved its interest and reliability (Aghababian et al., 2011). For the ToM evaluation, Maat et al. used the hinting task; a verbal test that required the subject to understand a hint in a dialog. In contrast, the present study used the V-SIR; here, the patient evaluated the likelihood of proposed intentions of the characters involved in short video excerpts, which showed complex social interactions. Arguably, each task involved different constructs within social cognition, like social perception, mental state inference, emotional empathy, etc., and these differences might explain the contrasting results.

Another explanation for the present results may be that there was no relationship between cognitive abilities and self-reported QoL. Studies on the relationship between cognitive performance and QoL have shown contradictory results. Boyer et al. (2012) found no significant statistical correlation between S-QoL 18 scores and neuropsychological measures of attention, memory, or executive functioning in a population of 113 patients with schizophrenia. Boyer et al. proposed that functional outcomes might be mediated through metacognitive capacities, particularly ToM abilities. The results of the present study did not provide evidence for this hypothesis. However, many metacognitive capabilities (insight, self-evaluation of performance, knowledge of our own cognitive strategies, etc.) cannot be assimilated into ToM measures, which by definition relates to the use of mental state concepts to interpret behavior, and that hypothesis may be accurate for restricted aspects of metacognition.

Although the present analyses did not identify significant correlations with S-QoL subscores, one item did correlate with the V-SIR score, but not the SCD score. This item was the ability to express one’s own feelings (S-QoL item No. 40). This isolated finding that needs to be taken cautiously suggests that the more severe the impairment in ToM abilities, the more difficulty the patient had in expressing his or her emotional states. A shortened version of the S-QoL, the S-QoL 18, which was designed with an item reduction procedure, includes this item (Boyer et al., 2010). The present result strengthened the relevance of this item, because it may capture a significant cognitive aspect of the pathology. Additional research is needed to evaluate this opinion.

### Limitations of the study

The present work had several limitations. First, the present population, selected for a clinical trial, may not be representative of the entire schizophrenic population. Moreover, some data were not available, including the age of illness onset, medication doses, educational levels, and neuropsychological assessment.

Another limitation was that we did not evaluate participants’ insight. Aghababian et al. (2011) have shown that insight of the mental illness is negatively linked with the same version of the S-QoL that we have used. In schizophrenia, the insight impairment is strongly associated with functional outcome (Tandon et al., 2010). Some studies have found a correlation between ToM and insight into illness (Bora et al., 2007; Langdon and Ward, 2009), and Pijnenborg et al. (2012) estimate that the affective component of ToM is an important contribution to the variance of the insight schizophrenia. Falissard et al. (2006) remarked that QoL self-evaluation may be influenced by defensive reactions. With a different measure, it was demonstrated that QoL remained stable, despite a decrease in symptoms (Wilson-d’Almeida et al., 2013). This stability might be due to the fact that improvement is associated with better judgment about one’s position in life and higher expectations. This suggested that QoL should not be considered the only perception of impairment. Dissatisfaction can also result from complex psychological and cultural processes. The evaluation of cognitive insight, also referred to as metacognitive skills, may be of interest in future research.

## Implications of the Study

The mental health care providers often base their therapeutic propositions on patient complaints, and they consider that the patient’s insight into his/her difficulties is a motivational factor that could predict therapy effectiveness. The present results and the literature have indicated that, although ToM impairments were related to objective symptoms and functioning, patients tended to have quite unreliable insight about the impairment. Consequently, it appears important to assess social cognition with objective, validated tools to provide the rationale for a proposal of therapy targeting these cognitive skills.

## Conflict of Interest Statement

Pr. Marie-Christine Hardy-Bayle received fees from Lilly France as the French coordinator of the study. Dr. Nadine Bazin received fees from Lilly for training the investigators on the V-SIR and SCD.
